# Wild pea (Pisum sativum L. subsp. elatius (Bieb.) Aschers. et Graebn. s.l.) at the periphery of its range: Zagros Mountains

**DOI:** 10.18699/VJ20.596

**Published:** 2020-02

**Authors:** O.E. Kosterin, V.S. Bogdanova, A.V. Mglinets

**Affiliations:** Institute of Cytology and Genetics of Siberian Branch of the Russian Academy of Sciences, Novosibirsk, Russia Novosibirsk State University, Novosibirsk, Russia; Institute of Cytology and Genetics of Siberian Branch of the Russian Academy of Sciences, Novosibirsk, Russia; Institute of Cytology and Genetics of Siberian Branch of the Russian Academy of Sciences, Novosibirsk, Russia

**Keywords:** Pisum sativum L. subsp. elatius (Bieb.) Aschers. et Graebn., Pisum sativum L. subsp. biflorum (Rafin.) Soldano, Lathyrus oleraceus L. subsp. biflorus (Rafin.) Coulot et Rabaute, pea, crop wild relatives, Iran, Zagros Mountains, Fertile Crescent, Pisum sativum L. subsp. elatius (Bieb.) Aschers. et Graebn., Pisum sativum L. subsp. biflorum (Rafin.) Soldano, Lathyrus oleraceus L. subsp. biflorus (Rafin.) Coulot et Rabaute, горох, дикие сородичи культурных растений, Иран, горы Загрос, Плодородный полумесяц

## Abstract

Characteristics of wild peas and their habitats at the periphery of the range are interesting with respect to their potential importance for pre-breeding programs aimed at selection for different environmental conditions. However, wild pea diversity in peripheral regions is insufficiently represented in the existing germplasm collections. In such regions, wild pea populations are rare, small in size and suffer from climatic change and land exploitation, hence their focused search is strongly desirable. A two-week-long expedition to Iran in May 2017 revealed two small populations of the wild pea (Pisum sativum subsp. elatius) in the Zagros Mts, in Aligudarz and Khorramabad Districts of Lorestan Province, Iran, at elevations of 1841 and 1971 m a.s.l., respectively. Their habitats are briefly described. Two pea accessions derived from them, CE9 and CE10, were characterised for some visible and molecular characters. These peas appeared to belong to the evolutionary lineage B, recognised by us earlier in P. sativum as opposed to the so-called lineage AC. They contain a unique non-conservative substitution in subtype 5 of histone H1 and turned to be most related to some wild pea accessions originating from southern and south-eastern Turkey and Golan Heights. Scarce information available on wild pea occurrence in Iran suggests their existence in the south-western principal slope of Zagros Mts and southern principal slopes of Elborz and Kopet Dagh Mts. It was found that wild peas representing the evolutionary lineage B produce poorly open and poorly coloured flowers (as reported by us earlier) only in the greenhouse conditions but normally pigmented and open flowers in the wild and mesh houses at open air in Israel. Some issues of pea taxonomy are discussed.

## Abstract (ru)

Местообитания и признаки диких сородичей гороха на периферии их ареала небезынтересны ввиду их возможного значения для селекции на соответствие различным условиям среды. В то же время разнообразие дикого гороха из периферийных регионов недостаточно представлено в мировых коллекциях генетического материала. На периферии ареала его популяции встречаются редко, невелики по размеру и страдают от землепользования и изменений климата, поэтому весьма желателен их целенаправленный поиск. В ходе двухнедельной экспедиции в Иран в мае 2017 г. были обнаружены две популяции дикого посевного гороха (Pisum sativum L. subsp. elatius (Bieb.) Aschers. et Graebn.) в горах Загрос, в районах Алигударз и Хоррамабад провинции Луристан, Иран, на высоте 1841 и 1971 м над уровнем моря соответственно. Приведены краткие описания их местообитаний. Полученные из этих популяций линии гороха, CE9 и CE10, охарактеризованы в отношении некоторых внешних и молекулярных признаков. Они принадлежат к эволюционной линии B, выявленной нами ранее и противопоставленной так называемой линии АС в пределах вида P. sativum, имеют уникальную неконсервативную замену в субтипе 5 гистона Н1 и наиболее родственны некоторым образцам из южной и юго-восточной Турции и Голанских высот. Имеющаяся в литературе скудная информация о местонахождениях дикого гороха в Иране свидетельствует, что он распространен в этой стране на юго-западном макросклоне гор Загрос и на южных макросклонах гор Эльбурс и Копетдаг. Обнаружено, что представители эволюционной линии В дикого гороха формируют плохо раскрытые и слабо пигментированные цветки в условиях теплицы, тог- да как в природе и сеточных растильнях на открытом воздухе в условиях г. Реховот, Израиль, они производят нормально раскрытые и пигментированные цветки. Обсуждаются некоторые актуальные вопросы таксономии гороха.

## Introduction

The pea (Pisum sativum L.) is an important crop of higher latitudes useful as vegetable, grain, fodder and natural soil fertiliser in crop rotation and was among the founder crops first domesticated in the Near East in the course of the so-called ‘Neolithic revolution’ (Zohary, Hopf, 2000; Weiss, Zohary, 2011). The cultivated pea was the first genetic object and has accumulated enormous genetic and phenotypic variation (Blixt, 1972; Makasheva, 1979; Kosterin, 2016a). At the same time, representatives of the same species, P. sativum, still exist in the wild, enjoying a broad range in the Mediterranean in the broad sense, stretching from Portugal in the west (ca 9° W) to Turkmenistan in the east (ca 60°30′ E) and from Normandy in the north (48°44′ N) to Sinai in the south (ca 34° N).

Taxonomical attribution of wild representatives of P. sativum was equivocal until some temporary stabilisation under a compromise system by Maxted and Ambrose (2001), who lumped all them under subspecies P. sativum L. subsp. elatius (Bieb.) Aschers. & Graebn. in a broad sense, defined solely by the fact of being wild and thus inevitably paraphyletic since the cultivated pea, P. sativum L. subsp. sativum, was derived from a wild representative of the same species. This treatment, however, disregards the fact that according to the rules of the botanical nomenclature (International Code…, 2012), the correct name of wild peas in a subspecies rank should be P. sativum L. subsp. biflorum (Rafin.) Soldano rather than P. sativum subsp. elatius (Soldano, 1992). Moreover, the comprehensive molecular phylogenetic analysis of the tribe Fabeae by Schaefer et al. (2012) suggested that the genera Pisum L. and Vavilovia A. Fed. form a branch inside the genus Lathyrus L. so making the latter paraphyletic. As a consequence, Coulot and Rabaute (2016) made an attempt to revise the Fabeae taxonomy to make it phylogenetically consistent and, in particular, downgraded Pisum to the section Lophotropis (Jaubert et Spach) H. Schaefer, Coulot et Rabaute of the genus Lathyrus L. Kosterin (2017) pointed out that the section name Lophotropis was incorrect and corrected its name to Lathyrus sectio Pisum (L.) Kosterin, which was accepted by Coulot and Rabaute (2017). Although phylogenetically consistent, this revised system (Coulot, Rabaute, 2016, 2017; Kosterin, 2017), where the pea gets the name Lathyrus oleraceus Lamarck and its wild representatives the name L. oleraceus Lamarck subsp. biflorus (Rafinesque) H. Schaefer, Coulot et Rabaute, is practically inconvenient as downgrading the small genera Pisum L. and Lens L. which contained such important crops as pea and lentil, respectively. Hence scholars whose interest to these two groups is motivated, at least to some extent, by practical agricultural aspects are either reluctant to adopt so radically revised a system or, more frequently, have no idea of it. In this paper we will keep to the habitual although somewhat outdated system by Maxted & Ambrose (2001) and denote wild representatives as P. sativum subsp. elatius.

As crop wild relatives, wild peas are practically important as a source of genetic diversity potentially valuable for pea breeding, first of all genes for resistance to various pests, diseases and draught (Kosterin, 2016b). No doubt, any information on the wild pea natural populations and their habitats is of importance, both theoretical and practical, i. e. for preliminary conjectures about selectively useful genes in a given population.

In spite of the great natural range of wild peas, in most parts of it they are rare plants with small populations (Maxted, Kell, 2009), strongly affected by sheep and goat grazing and, supposedly, by global warming (Coyne et al., 2011). Information on their habitat and ecology is scattered over local floras where it is provided in few general words at most. At the same time, it may be useful for at least a preliminary evaluation of usefulness of particular wild pea stocks for pre-breeding focused on certain traits. Two works from Israel, nearly the core of diversity of wild peas and the country where they are perhaps most common, contain more detailed information. Ben-Ze’ev and Zohary (1973) provided information on the habitat from where each wild pea accession involved into their study originated. Abbo et al. (2008) provided most detailed information on Israeli habitats of wild peas including the rock and soil types. Zlatcović et al. (2010) characterised the habitat and population of P. sativum subsp. elatius at the Pčinja River in SE Serbia.

We found populations of the wild pea subspecies P. sativum subsp. elatius in a number of regions at the periphery of its range. For the time being these are, from west to east, Portugal, Crimea, the Caucasus within Krasnodarskiy Kray, and Iran. The information on the wild pea habitat and population found in NE Portugal is published in ‘Materials and methods’ in Zaytseva et al. (2015) and a photo of a withered pea plant of that population is published in Kosterin (2016b, Fig. 1). This paper concerns the wild pea findings in the Zagros Mts in Iran. Populations of crop wild relatives from Zagros are of special interest since these mountains are considered the eastern part of the so-called Fertile Crescent, the area of origin of the ‘Neolithic Revolution’ in the Near East (Zohary, Hopf, 2000).

**Fig. 1.**
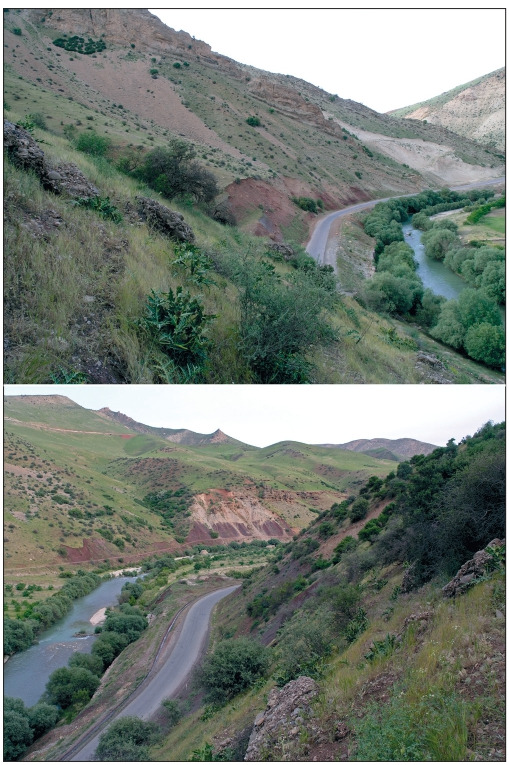
The habitat of wild peas in Iran, Ostan-e Lorestan, Shakhrestan-e Aligudarz, Bakhsh-e Besharat, at Kagelestan-e Bar Aftab village.

On May 18–31, 2017, the first author had an opportunity to join a dipterological expedition to Iran focused at long-legged flies (Dolichopodidae) by Igor Y. Grichanov from All-Russian Institute of Plant Protection, Saint-Petersburg, Russia, and Azam Ahmadi from Baran Plant Protection Institute, Arak, Ostan-e Markazi, Iran. The entomological results were published in Grichanov et al. (2017) and Kosterin, Ahmadi (2018); the latter source contains detailed descriptions of the localities examined.

## Materials and methods

**Search for wild pea populations.** The expedition was based at Arak City, the capital of Markazi (Central) Ostan (province) and visited Markazi, Lorestan and Esfahan Ostans. In total, 33 localities were examined, mostly associated with running water (because of the entomological focus). Four times, on May 23, 25, 26 and 31, the first author had an opportunity to visit Lorestan and to examine the first outposts of the Mediterranean vegetation in the valleys of the Higher Zagros (the peaks of which were still covered by snow). The Silakhor Plain lying north-west of the Higher Zagros is in its rain shadow and both the valley itself (except for the floodplains of few rivers) and the bordering mountain slopes lack natural arboreal vegetation (only that associated with human activity is present). At the same time, in the upper parts of the valleys dividing the south-western slope of the Higher Zagros at ca 2000 m above sea level (a.s.l.), an open stand of the Persian Oak (Quercus brantii Lindl.) appears, with the participation of the Montpellier Maple (Acer monspessulanum L.), Christ’s Thorn (Paliurus spina-christi Mill.) and Prunus sp. At lower levels of those valleys this oak parkland covers the slopes entirely. The first author examined four valleys and found wild pea populations in two of them.

**Plant growing and derivation of wild pea accessions.** Seeds collected in nature in two populations found in Iran (at Kagelestan-e Bar Aftab and Istgah-e Bisheh, see below) were sowed in the greenhouse in autumn generation (October–December) 2017. One plant from each of these two populations was chosen, their progenies were propagated in the same greenhouse in spring generation (February–May) 2018, and gave rise to accessions CE9 and CE10, respectively.

The prefix ‘CE’ was at first introduced by us for wild pea accessions derived from wild populations found in 1991 in Crimea (Kosterin, Bogdanova, 2008), abbreviated from ‘Crimean elatius’. It appeared convenient to adopt it for the entire collection of confirmed wild peas of our Laboratory of Genetics and Evolution of Legumes at ICG SB RAS with its meaning reconsidered as certa exempla, that means ‘true, reliable specimens’ in Latin. (The above-mentioned wild pea accession derived from a population from NE Portugal, published as “PE1” (Zaytseva et al., 2015, p. 236), with a synonym JI3557 in John Innes Centre collection, gets in this system the accession number CE11.) This collection is a part of GENAGRO collection at this institute.

**Molecular procedures.** DNA isolation, PCR and CAPS analysis of the plastid gene rbcL and mitochondrial gene cox1 were carried out as described in Kosterin, Bogdanova (2008). The nuclear gene His5 of histone H1 subtype 5 was sequenced according to Zaytseva et al. (2012); the plastid spacer psbAtrnH, according to Zaytseva et al. (2017).

DNA sequences obtained in these works are stored in public databases with the following accession numbers: MK933283, MK933284 (psbA-trnH spacer), MK952766

## Results

**Natural populations of wild pea (Pisum sativum subsp. elatius s. l.) in Zagros**

**Fig. 2.**
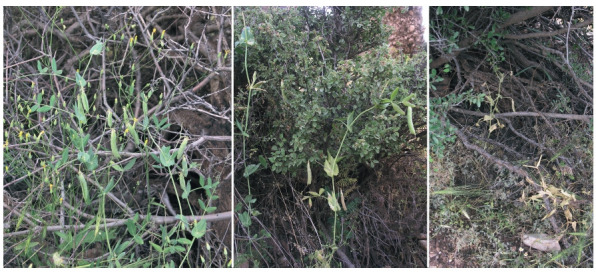
Plants of P. sativum subsp. elatius of the population at Kagelestan-e Bar Aftab village.

A wild pea population was found on May 23, 2017 in Iran, Ostan-e [Province of] Lorestan, Shakhrestan-e [County of] Aligudarz, Bakhsh-e [District of] Besharat, 700 m N of the centre of Kagelestan-e Bar Aftab village, at 33°02′13″ N, 49º39′23″ E, 1841 m a.s.l. The wild peas were found in the lower part of the steep NW slope of the left (opposite to the village) board of the valley of the Rudbar-e Aligudarz River (a Dez River tributary). The slope had large rock (supposedly dolomite) outcrops and was covered with annual Graminea vegetation and sparse bushes of a wild almond Prunus scoparia Schneider (Fig. 1). About 40–50 plants were found on an area ca 10 × 10 m at the bases of spiny almond bushes seemingly protecting them from being grazed by cattle, the paths of which were numerous on that slope. The plants were at the final vegetation stage, with the vegetative parts withered, pods ripen, about half of them dried out and about quarter of them dehisced (Fig. 2). In total 193 seeds were collected, 48 of which later appeared to be infested by the pea weevil (Bruchus pisorum L.). One of those seeds gave rise to accession CE9 (= W6 56889 in the USDA GRIN).

In the same habitat and also at the almond bush bases another crop wild (distant) relative, Cicer anatolicum Alef. occurred, at the stage of flowering and young pods. (The unripen seeds were collected and later sowed in the greenhouse, one plants emerged but too late to be allowed to produce seeds, a DNA sample being isolated from it.)

**Fig. 3.**
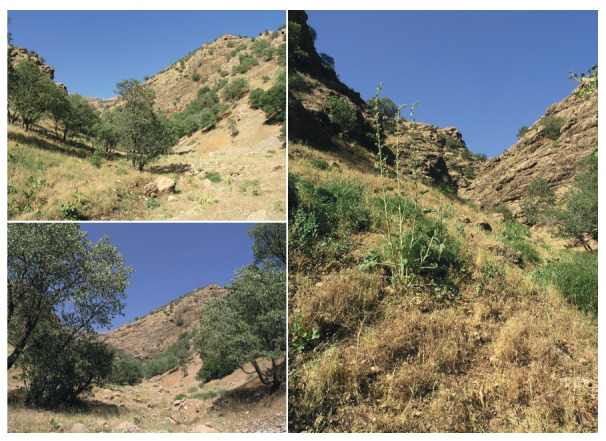
The habitat of wild peas in Ostan-e Lorestan, Shakhrestan-e Khorramabad, Bakhsh-e Papi, 6.7 km NW of Istgah-e Bisheh village.

**Fig. 4.**
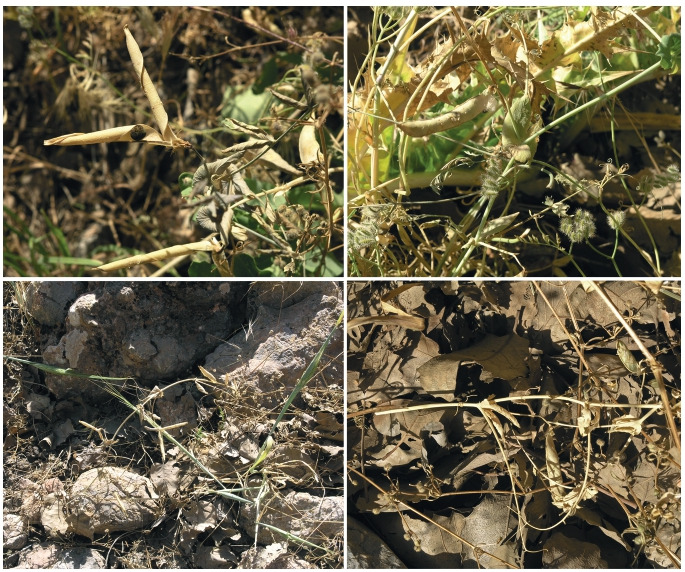
Plants of P. sativum subsp. elatius of the population at Istgah-e Bisheh village.

The second wild pea population was found on May 31, 2017 in Iran, Ostan-e Lorestan, Shakhrestan-e Khorramabad, Bakhsh-e Papi, 6.7 km NW of Istgah-e Bisheh village (broadly known as simply Bisheh), 33°22′12″ N, 48°49′34″ E, 1971 m a.s.l. (this is 85 km NW of the previous locality), in a rocky dell with a stony/detritous bottom with rock outcrops, its upper part becomes a small gorge between large cliffs (Fig. 3). The vegetation was dry Persian Oak stand; annual Poaceae, already withered, predominated in the grass layer. Wild pea plants occurred on the detritous bottom and at the base of the rocky right slope of the dell, in a stripe ca 110 m long but not more than 10 m wide; they alternate with plants of some perennial vetch (Vicia sp.). Not less than a hundred plants were found. They were completely withered (Fig. 4), with all normally developed pods already open, with on average one seed per plant found captured in the rolled pod walls (see Fig. 4, top left). Only some pods which dried underdeveloped, had the walls not opened. Also about a dozen of ripen but not yet dried pods were found, bearing traces of pea weevil eggs, traces of burrowed young larvae (up to 20 per pod), and solitary neoplastic pustules caused by the Np gene (see Fig. 4, top right) and being the plant’s defense reaction to weevil oviposition (Berdnikov et al., 1992). In total 87 seeds were collected, of them 24 appeared infested by the pea weevil. One of those seeds gave rise to accession CE10 (= W6 56890 in the USDA GRIN).

It should be noted that on May 25, 2017 in the environs of Hayan village (33°47′ N, 48°54′25″ E, 1644 m a.s.l.) (Shakhrestan-e Borujerd), also in Lorestan but at the NE foothills of the Inner Zagros Range facing the Silakhor Plain, in a roadside herbaceous vegetation under a stripe of poplars, the first author found several plants of obviously feral peas escaped from cultivation. They had two small non-dehiscing pods (phenotype dpo; the character of cultivated peas) per inflorescence, seeds with anthocyanin coloration (phenotype Fs), marble pattern (phenotype M), and non-gritty testa (phenotype gty). Locals told that in that place peas had been grown for fodder 7–8 years ago.

**Characters of wild peas from Zagros**

We scored accessions CE9 and CE10 for some molecular characters involved in our previous studies. Both lacked the recognition sites for HspAI endonuclease in the plastidic rbcL and the site for PsiI restriction endonuclease in the mitochondrial cox1 gene. The sequence of psbA-trnH plastidic spacer of CE9 was identical to its consensus in peas (Zaytseva et al., 2017) while that of CE10 had a substitution A→T in position 128. The sequences of the His5 gene coding for histone H1 subtype 5 obtained from CE9 and CE10 were compared to those obtained in the course of our earlier works (Zaytseva et al., 2012, 2015; Bogdanova et al., 2018). The His5 sequences in CE9 and CE10 appeared identical to each other and most close to those of JI1794 (Golan Heights) and P012 (Turkey, Adiyaman Province) (also identical to each other), differing from them only by an A→C substitution in position 452 resulting in a non-conservative substitution of lysine to threonine in the globular domain of the molecule (protein position 111). This substitution was not found in any other pea accession. One more His5 sequence very close to the above mentioned, that from accession JI3233 (Syria), differed from them by the T→C substitution in position 722 leading to the valine→ alanine amino acid substitution in the C-terminal domain.

**Fig. 5.**
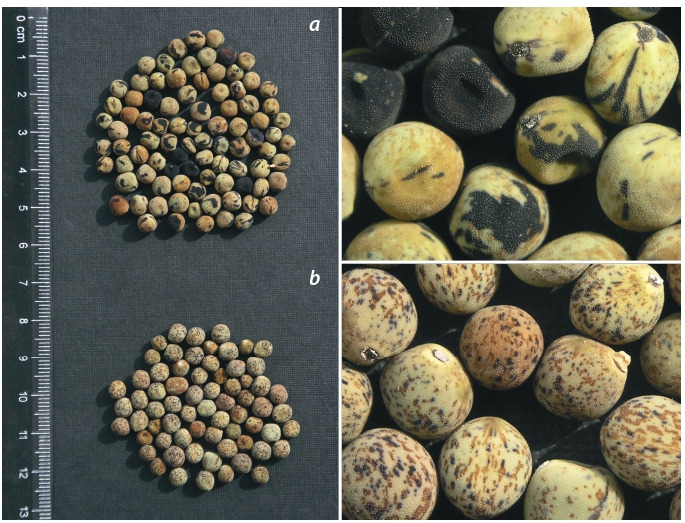
The seeds of wild pea accessions CE9, originating from a population at Kagelestan-e Bar Aftab village (a), and CE10, originating from a population 6.7 km NW of Istgah-e Bisheh village (b).

The seeds of both accessions CE9 and CE10 (Fig. 5) have gritty testa (phenotype Gty, a wild character), black hilum (Pl), a brownish marble pattern (M), violet specks (Fs), no furca pattern (rf). Those of CE9 in addition have conspicuous violet stripes (Ust) (see Fig. 5, a). The young seed ground colour is rather pale greenish-grey in CE9 (see Fig. 5, a) and brownish-grey in CE10 (see Fig. 5, b); the shape is slightly irregular, not perfectly sphaeric. If scarified and sowed after 1–2 month after formation, they readily germinated, earlier than most other peas.

**Fig. 6.**
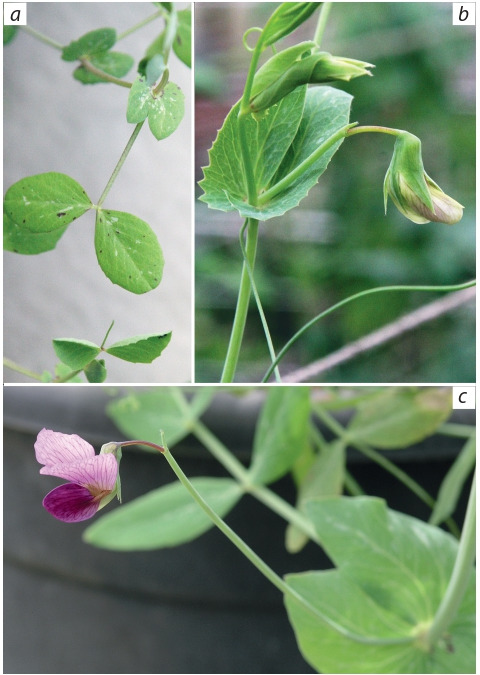
Pea accession CE9: leaflets and stipulae of a young plant (a) and flowers (b) in the ICG hydroponic greenhouse and in an outdoor mesh house at Agronomy Faculty of Hebrew University of Jerusalem, Rehovot, 07.05.2019 (c).

The plants found in the Kagelestan-e Bar Aftab population (CE9) in nature were up to 70 cm in height, those in the Bisheh population (CE10) only up to 40 cm. In the intraspecies taxonomy of wild representatives of P. sativum used in the past and based on the plant height, they would be classified as ‘Pisum humile’ (Ben-Ze’ev, Zohary, 1973) or P. sativum subsp. syriacum Berger (Makasheva, 1979). In the conditions of the greenhouse spring vegetation the difference in plant height retained but had a lesser magnitude of ca 30 %: the CE9 plants were 69–122 cm tall (mean 106.0 ± 13.2; n = 39), flowered from 14th–19th node (mean 16.7 ± 1.0) and totally had 18–23 nodes on the main stem (mean 21.0 ± 1.2); the same parameters of CE10 had the following values: 40–94 (mean 74.1 ± 16.7; n = 37), 14–18 (mean 15.7 ± 1.0) and 17–21 (mean 19.2 ± 1.1), respectively. The plants were elegant, with long internodes, narrow stipulae only moderately dentate at base, rather narrow rhomboid-oval very slightly dentate leaflets (larger in CE10), had numerous aerial cameras on both stipulae and leaflets and outer anthocyanin rings (but no inner rings) at the base of stipulae (manifestation of some of the dominant alleles of the gene D). Besides, CE9 (but not CE10) had tiny but conspicuous violet specks on leaflets (Fig. 6, a). The plants moderately branched at the base and the main stem below flowering nodes, had medium-long peduncles, with one or two (at the middle of the range of flowering nodes) flowers. The pods dehisced explosively upon ripening (phenotype Dpo, a wild character); their walls bore sparse and small neoplastic pustules caused by the Np gene in the greenhouse conditions (while in the wild as a response to the weevil oviposition) (Berdnikov et al., 1992).

**Fig. 7.**
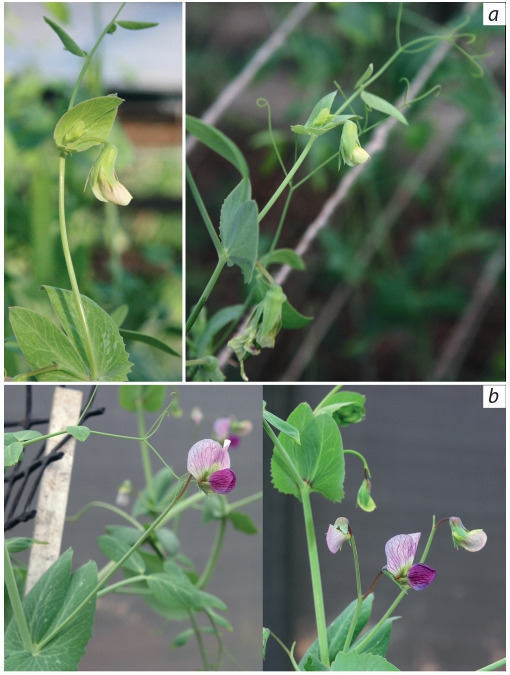
Flowers of pea accession CE10 in the ICG hydroponic greenhouse (a) and in a mesh house at Agronomy Faculty of Hebrew University of Jerusalem, Rehovot, 07.05.2019 (b).

The flowers of both Zagros accessions grown in the greenhouse never opened fully and were greenish (Figs 6, b, 7, a). At the same time in the natural-like conditions of an outdoor mesh house at Agronomy Faculty of the Hebrew University of Jerusalem in Rehovot, Israel, these accessions provided large, fully open and well coloured flowers (Figs 6, c, 7, b).

## Discussion

**Wild peas in Iran**

Most part of the huge territory of Iran is in the rain shadow of Zagros Mts, which captures precipitation from the Mediterranean, and hence is too arid for wild peas. The Iranian Plateau and the north-eastern (inner) principal slope of Zagros have mostly Irano-Turanian rather than Mediterranean vegetation, mostly its desert versions (Zohary, 1973). The Mediterranean vegetation and flora, to which wild peas belong, is present only in the sea-facing outer principal slopes of the Iranian mountain systems: the south-western slope of Zagros Mts and the northern slope of Elborz Mts. These are the regions where wild pea populations should be sought for. (According to observations by the first author, the vegetation change is obvious when crossing the Higher Zagros Range in Lorestan. In the south-west of this province the most widespread vegetation is the Persian Oak (Q. brantii ) parkland extending to ca 2000 m a.s.l., while in the north-eastern part any natural arboreal vegetation is missing even as low as at 1700 m a.s.l.) The presence of wild peas in the Iranian southern slope of Kopet Dagh is also possible but is probably marginal in those rather hostile arid mountains.

Reports of wild peas from Iran are found in two multivolumed Floras: “Flora Iranica” published in Latin/English and “Flora of Iran” published in Farsi; curiously, data in these reports do not overlap. The volume devoted to the tribe Vicieae (currently Fabeae) (Reichinger, 1979) contains seven locations for P. sativum subsp. elatius: three in Lorestan in western Iran (Bisheh, 2100 m a.s.l. [near which but at 1971 m a.s.l. CE10 was collected by us]; “Dou Rud” [Dorud]; Shah-Bazan, 600 m a.s.l., but the latter is presently in Khuzestan Ostan) and four in northern Iran, one in Gorgan Province (now Golestan Province) (Ziarat env.) and three in Gilan Province (Bandar- e Pahlavi (now Bandar-e Anzali); Lake Mordab westerly of Bandar-e Pahlavi, 26 m a.s.l.; Astara). The 33th volume of “Flora of Iran” (Pakravan et al., 2000) contains different localities for the same taxon: one in Ostan-e Kurdistan (Sanandaj, 1380 m a.s.l.), three in Ostan-e Azerbaijan (Arasbaran, 63 m a.s.l.; Sardasht; Ighon, 1200–1500 m a.s.l.), one in Ostan-e Golestan (Gombat, 750 m a.s.l.) and two in Ostan-e Fars (Nurabad and Doshman Ziari, 1800 m). Occurrence of wild peas as southerly as Fars, if true, was unexpected.

Both sources do not report for Iran P. sativum subsp. elatius var. pumilio Meikle or its synonyms. At the same time the map in Maxted, Kell (2009, Fig. 18) shows in western Iran five localities of P. sativum subsp. elatius var. pumilio but none of P. sativum subsp. elatius var. elatius. As the source of information for that map an unpublished thesis by A.S. Mumtaz is indicated, which was defended in 2005 in Birmingham University. Most probably here we face an equivocal treatment of intra-species taxonomy of P. sativum by different authors.

World germplasm collections hitherto contained only four accessions claimed to represent wild peas from the huge territory of Iran. Accession IG65050 (Iran, Lorestan, 33.667° N 48.55° E) is from ICARDA collection and originated form Zagros Mts. The coordinates adduced refer to a south-western slope at 1800 m a.s.l. in the northern environs of Beyravand- e Jonubi village in Khorramabad Shahrestan. Accession PI143673 was derived from plants collected in 1940 in Dorud, Lorestan. For accession JI1030 (=PI140295), coordinates 34° N and 56° E and provenance “Khorassan” were indicated, but the coordinates are most probably erroneous as referring to the low and deserted Tebess Mts between Dash-e Kavir and Dasht-e Lut Deserts in South Khorasan Province. (It is not excluded that the coordinates were arbitrarily indicated for the centre of the historical Khorasan region.) For accession JI2105 (ITPDB 104333, = PI227258) only coordinates 32.659° N, 51.671° E are provided by the online database of John Innes Centre collection; they refer to the Esfahan City environs. However, we grew out the latter accession and found it to represent a cultivated pea with non-dehiscing pods (Kosterin et al., 2010). The two untested accessions from Lorestan perhaps represent true wild peas. At least their provenance is close to our findings: CE9 was collected 120 km SE and CE10 43 km SE of Beyravand-e Jonubi, the presumed provenance of IG65050.

Thus, wild peas are reliably known in Iran from the southwestern principal slope of Zagros and the western and eastern parts of Elborz, although in general Iran plus Turkmenistan occupy about one third of the Pisum natural range by longitude. It is broadly accepted that in the western Eurasia, productive farming arose and plant domestication occurred in the so-called Tauro-Zagros Arch or Fertile Crescent, a mountain belt including Golan Heights, Taurus and Anti-Taurus Ranges and Zagros Mts (Zohary, Hopf, 2000). Formally the Zagros Mts comprise about one third of the Arch and most of these mountains are in the territory of Iran, from which they only protrude to the Iraqi Kurdistan. However, only the south-western principal slope of Zagros has the Mediterranean vegetation and can be attributed to the Fertile Crescent. Based on literature, the southern border of the wild pea range in Iran can be extrapolated to cross Ostan-e Fars.

**Wild pea habitats**

The CE10 locality is 75 km NW and the CE9 locality 47 km SE of the Oshtorankuh Mountain (4050 m a.s.l.), the highest summit of Lorestan. At the same time, the mountains situated to the south and south-east of these localities do not exceed 2000–2500 m a.s.l. Thus the wild pea habitats found are not situated in the main rain shadow of the Higher Zagros, that is the reason of the appearance of arboreal vegetation of the Mediterranean type, which is absent to the north and north-east of them at any elevations. One can note that all wild pea findings in Lorestan were made at close elevations a. s. l.: 2100 m (Reichinger, 1979), 1971 m, 1841 m (our findings) and 1800 m (reconstructed from coordinates of accession IG65050). The former value refers to the upper limit of arboreal vegetation in this area.

Occurrence of wild peas in oak parkland was expectable since this is one of the primary habitats of at least some wild pea ecotypes elsewhere, e. g. in Israel (Ben-Ze’ev, Zohary, 1973; Abbo et al., 2008). It remains unclear if a slope with spiny almond bushes is a regular habitat of wild peas (and C. anatolicum) in Iran since the first author failed to examine more examples of such habitats.

The analysis of regional literature suggests that wild peas are associated with calcareous habitats on limestone or dolomites throughout their vast range but also with igneous rocks and volcanic slag in Israel (Ben-Ze’ev, Zohary, 1973; Abbo et al., 2008) and East Turkey (Abbo et al., 2013). The Higher Zagros is composed mostly by the Mesozoic dolomites that conforms the notion of predominant calciphily of P. sativum subsp. sativum.

**Relationships of Zagros wild peas**

Such characters as dehiscing pods (Dpo) and gritty seed testa (Gty) evidence that both small populations found among Mediterranean vegetation represent genuine wild peas. Absence of the target restriction sites in the plastidic rbcL and mitochondrial cox1 genes (Kosterin, Bogdanova, 2008) in CE9 and CE10 suggests their belonging to the so-called evolutionary lineage B of wild P. sativum. This lineage was revealed by us earlier, as a monophyletic clade opposed to the so-called lineage AC, in the phylogenetic reconstruction based on the histone H1 genes (Zaytseva et al., 2012, 2015, 2017) and plastid genomes (Bogdanova et al., 2018) and is identifiable by convenient molecular markers from different cellular genomes (Kosterin, Bogdanova, 2008; Kosterin et al., 2010). The lineage B includes a great number of wild peas as well as the cultivated pea subspecies (P. sativum subsp. sativum). At the same time, as expected for wild peas, CE9 and CE10 had no 7-bp deletion in the plastid psbA-trnH spacer which, with only two known exceptions, is specific to (a synapomorphy of) the cultivated subspecies (Zaytseva et al., 2017). The A→T substitution in position 128 of this spacer in CE10 is shared by wild pea accessions JI1794 (Golan Heights) and P017 (Turkey, Mersin Province) (unfortunately, this substitution was not mentioned by Zaytseva et al. (2017)). The His5 gene of both CE9 and CE10 contains the same nucleotide substitution resulting in the lysine→threonine replacement in the globular domain not found in any other pea accession; otherwise their His5 sequence is identical to those of accessions JI1794 and P012 (Turkey, Adiyaman Province). One more very close His5 sequence, differing from the above mentioned ones in one substitution, belongs to accession JI3233 (Syria).

We may conclude that the wild peas found by us in Zagros represent some subtle evolutionary branch of the lineage B also occurring at least in southern and southeastern Turkey, Golan Heights and Syria. It is noteworthy that JI1794 is a low plant, P017, CE9 and CE10 are moderately high and JI3233 is a high plant, which once again stresses the inapplicability of plant height to evaluate relatedness of wild peas (Ben-Ze’ev, Zohary, 1973). Also some genetic difference already found between CE9 and CE10 originating from the populations 85 km apart is noteworthy: the substitution in position 128 of psbA-trnH in CE10 and the violet stripes (Ust) on the seed testa, violet specks on leaflets and a greater plant height in CE9.

**Flowers of peas of evolutionary lineage B**

**Fig. 8.**
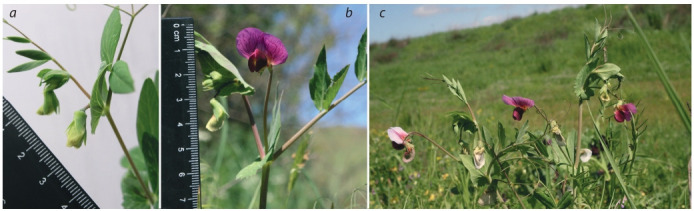
Flowers of the pea accession JI1794 originating from Tell Abu Nida Hill in Golan Heights in the conditions of the ICG hydroponic greenhouse (a) and in the wild population of Tell Abu Nida, 11.05.2019 (b, с).

Zaytseva et al. (2017) claimed that most of wild peas of the evolutionary lineage B have flowers poorly pigmented and opened, in contrast to well open and coloured flowers of cultivated peas, belonging to the same lineage, and wild peas of the ‘evolutionary lineage AC’. It turned out that this statement is true only of our greenhouse conditions. Accessions CE9 and CE10 grown in our greenhouse and in the mesh house in Rehovot, where the conditions were close to natural, have, respectively, poorly open greenish (see Figs 6, b, 7, a) versus well open, coloured and large (see Figs 6, c, 7, b) flowers. Analogously, the photos taken by S.A. Litvinskaya (pers. comm.) of wild peas of the lineage B in two populations at the Black Sea Coast of Krasnodarskiy Kray, Russia, show large, well open and coloured flowers, while the flowers of the plants grown in our greenhouse from seeds from these populations are poorly opened and coloured, as in the case of Iranian wild peas. Finally, accession JI1794 originating from the Tell Abu Nida Hill, Golan Heights, northern Israel, also produced poorly open greenish flowers in the greenhouse (Fig. 8, a), while plants in the natural habitat at Tell Abu Nida have well opened and strikingly coloured flowers, deep purple including the standard, most saturated among peas (Fig. 8, b, c). (In fact, some representatives of the lineage AC also produce poorly open and less coloured flowers in our greenhouse, e. g. accessions WG 26109 (Georgia), Pe 013 (Turkey) and sometimes also JI1096 (Greece) and JI3557 (Portugal).)

The factor that prevents the normal development of the flower corolla of wild representatives of the linage B (but not affecting the pods) in our greenhouse is still unclear. Our preliminary experiment ruled out the involvement of the temperature of germination. An edaphic factor can also be excluded, since the Black Sea natural habitats of wild peas are on limestone while the Tel Abu Nida habitat is on the basalt and volcanic ash. For the time being the temperature and illumination regime at the onset of flowering is the most probable candidate.

## Conclusion

In this paper we report findings of two wild pea population in the Zagros Mts comprising the eastern part of the Fertile Crescent which is considered to be an area where the founder crops were domesticated and the productive farming appeared in the Near East. Indeed, these populations represented the same evolutionary lineage of the species P. sativum to which the cultivated pea also belongs. However, another result of this study is the observed paucity of wild peas in these mountains and their occurrence only in their south-western principal slopes which offers suitable habitats. This is in contrast to their frequent occurrence and diversity in the western (e. g. Israel) and northern (SE Turkey) parts of the Fertile Crescent.

## Conflict of interest

The authors declare no conflict of interest.
